# Supplementary material for: Comparative multiomics analysis of cell physiological state after culture in a basket bioreactor
Source: Sci Rep. 2022 Nov 23;12:20161. doi: 10.1038/s41598-022-24687-4 (PMC9686226; doi:10.1038/s41598-022-24687-4)
Supplement: Supplementary file 1 — Supplementary Information 1. [file 41598_2022_24687_MOESM1_ESM.zip › raw data/Metabolomics raw data/4.MetDiffAnalysis/EG.vs.CG/ROC_neg/Com_585_neg_ROC.pdf]

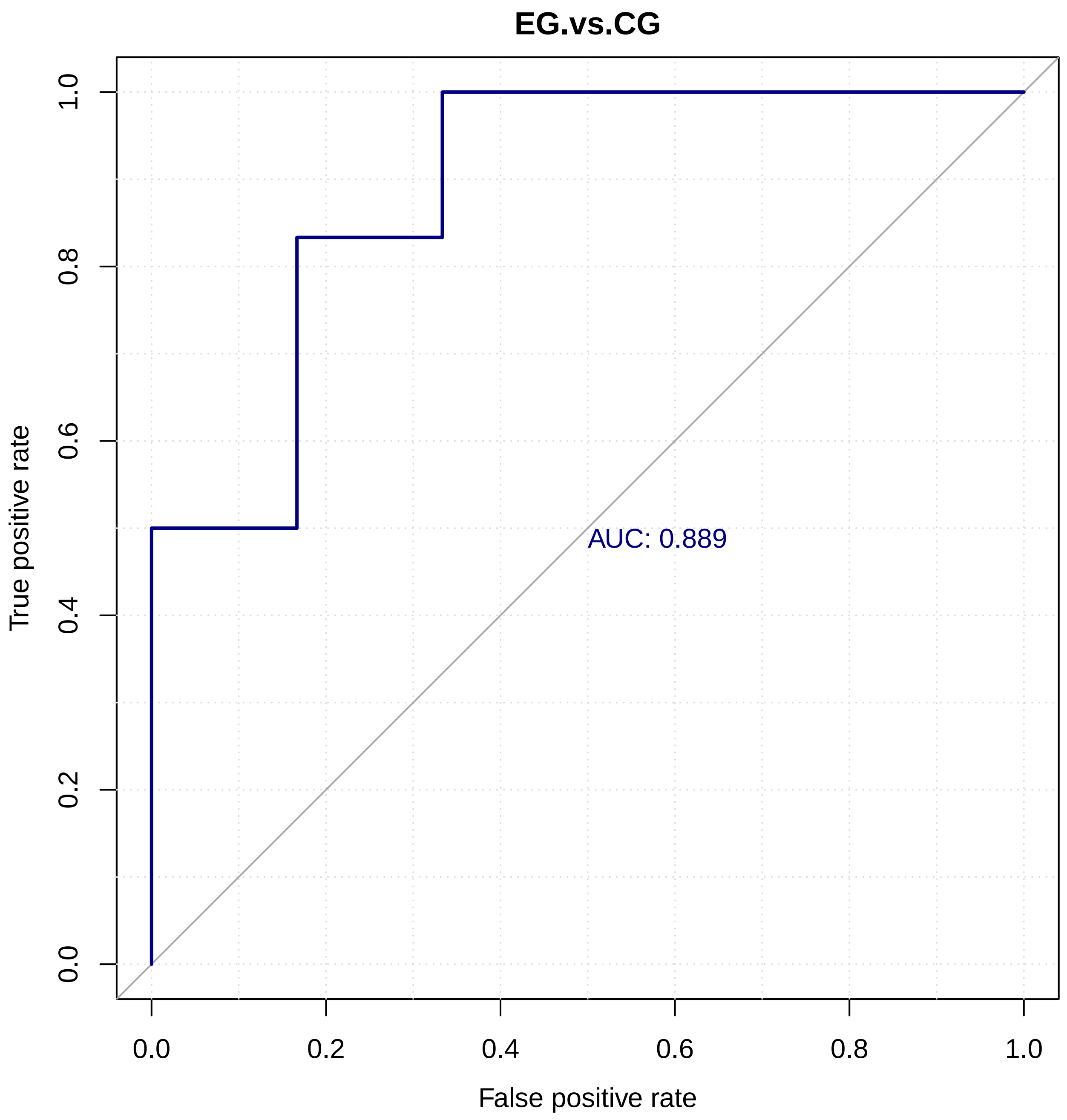
EG.vs.CG
True positive rate
0.0
0.2
0.4
0.6
0.8
1.0
False positive rate
0.0
0.2
0.4
0.6
0.8
1.0
AUC: 0.889
